# A novel way to optimize the process parameters by integrating the grey relational coefficient and the combined compromise solution for machining the CFRP composites

**DOI:** 10.1038/s41598-025-18368-1

**Published:** 2025-10-06

**Authors:** K. Shunmugesh, Arun Raphel, Sony Kurian, Shivashankarayya Hiremath, Gajanan Anne, B. R. N. Murthy, H. M. Vishwanatha

**Affiliations:** 1 Department of Mechanical Engineering, Viswajyothi College of Engineering and Technology, Ernakulam, 686670 Kerala India; 2 Department of Mechanical Engineering, Faculty of Engineering and Technology, SRM Institute of Science and Technology, Vadapalani Campus, Chennai, 600026 Tamilnadu India; 3 Department of Electrical Engineering, Viswajyothi College of Engineering and Technology, Ernakulam, 686670 Kerala India; 4 Department of Mechatronics, Manipal Institute of Technology, Manipal Academy of Higher Education, Manipal, India; 5 Department of Mechanical and Industrial Engineering, Manipal Institute of Technology, Manipal Academy of Higher Education, Manipal, India

**Keywords:** CFRP, Grey relational analysis, CoCoSo, CRITIC, Surface roughness, Composite industry, Mechanical engineering, Materials science, Composites

## Abstract

The quality of carbon fiber-reinforced plastic (CFRP) machining during wet drilling is strongly influenced by the moisture content and cutting tool geometry. The current investigation aims to determine the optimum drilling process parameters for machining CFRPs by combining the grey relational coefficient with the combined compromise solution (Grey-CoCoSo). A distance correlation-based criterion importance through intercriteria correlation (D-CRITIC) method was used to ascertain the weights of decision-making to manage the responses from multiple-measure decision-making. Several multiresponse outputs—the material removal rate (MRR), surface roughness (Ra), and delamination factor (DF)—were taken into consideration during the analysis of the input factors—the spindle speed (N), drill diameter (D), and feed rate (F). An enhanced MRR and reduced Ra and DF were achieved due to the optimal parametric conditions of D, F, and N when the D-CRITIC weight was set to 6 mm, 0.1 mm/rev, and 7500 rpm, respectively. As a result, the Ki value would have improved by 17.684%. Thus, N was found to be a pivotal parameter influencing MRR, DF, and Ra. The outcomes indicate that the proposed synergistic approach is beneficial for solving situations in which multicriteria decision-making is applicable to CFRPs. Compared with other conventional multicriteria decision-making approaches, the proposed newer approach is versatile and efficient in excluding variation. Thus, the grey-CoCoSo approach can be a promising way of optimizing process parameters for machining CFRPs.

## Introduction

Recently, carbon fiber-reinforced polymers (CFRPs) have been of great interest to the aeronautical, aerospace, and automotive industries owing to their excellent performance^1^. The use of CFRPs in these industries requires secondary processing, especially machining. Industries have witnessed significant advances in drilling and machining CFRPs, viz., fiber laser drilling, abrasive waterjet drilling, and classic drilling^2^. The conventional drilling technique is easy to use, economical, and somewhat efficient. Optimizing the process parameters can be a great advantage in making the machining of CFRPs more economical. An examination of the input components that impact product quality and guarantee the processing process’s output assessment standards has been reported^3^. Furthermore, the accuracy of the tolerance of the bore diameter, periphery geometry, delamination and uncut fibers, and surface roughness (Ra) are reported. The reported delamination is an indication of a shift in the thrust force from the centerline toward the periphery of the drill, which is due mainly to the effects of the drill geometry and the thrust force action. It is also possible to increase the feed rate without compromising delamination^4^. Delamination can be reduced, and Ra can also be enhanced by using a multifacet drill^5^. Few studies have reported the effects of soaking and the use of a variety of machining fluids during machining. Samples soaked for approximately 8 weeks at 23–50 °C resulted in reduced prejudice. Machining fluids such as Cindolube V30 ML, Hocut (GR 3000 and 795 B), and Metalina B800 are effective^6^. One emerging area of decision-making theory for workers of all professions is MCDM. The term continuous MCDM techniques in these definitions are named as MODM techniques^7^. The optimal value is equal to a quantitative value, and it can be obtained through continuous MCDM. MADM methods are discrete MCDM techniques^8^. Because of time constraints and/or the problem size and dimensionality, most of the time, providing a deterministic measure for transmitting expertise becomes impractical; consequently, most MCDM instances are intractable^9,10^.

Several studies have reported the use of computational, machine learning-based optimization of process parameters for machining CFRPs. The input process parameters for the drilling of composites reinforced with coir fibers are optimized via Nelder‒Mead and a genetic algorithm. Using these parameters, optimized parameters for torque, thrust forces, and tool wear were obtained considering the input parameters D, F, and N^11^. To quantify the geometrical parameters of the drill tool, a Taguchi experimental plan with parameters such as the brad center, helicoidal, reamer, and step has been executed. The torque, delamination, and thrust force respond optimally to response surface methodology^12^. A mixed approach of combining multiresponse optimization with principal component analysis and the fuzzy inference system-based Taguchi method has been explored to optimize delamination, the thrust force, and the torque factor for drilling CFRP plates^13^. Recently, machining parameter optimization techniques—particle swarm optimization (PSO)^14,15^, the whale optimization algorithm^16^, the preyfish optimization algorithm^17^, fuzzy logic^18^, response surface methodology^19^, and neural networks^20^—have been reported. However, these methods are labor intensive; hence, there is a scope for exploring better approaches to solve multicriteria policymaking problems.

Grey relational analysis (GRA) is also a widely used technique for optimizing multiple input factors to achieve the optimal response of the output factors^21,22^. It is a useful method for handling dark, low-quality, and insufficient data^23^ that are considered grey data. To produce a more favourable response output, fuzzy logic theory was stretched into a structure with additional variables, thus improving the GRA^24,25^. In a given supply chain, the outlier supplier was identified by replacing the CoCoSo method with a synergistic approach of the normalized weighted and the normalized weighted geometric Bonferroni mean functions. Flexible decision-making is made possible by the CoCoSo model, which considers the interplay between several input qualities^26^. A novel CoCoSo technique that uses T-spherical fuzzy sets to solve multiple attribute group decision-making issues by utilizing Frank operational rules and the Softmax function^27^. The CoCoSo approach is a powerful tool for determining the optimal aim by combining three aggregation algorithms with Shannon’s entropy. The process involves 2 techniques—the entropy technique, in which the material weights of the criteria are calculated, and the CoCoSo technique, in which the good material options are ranked^28^. The simplicity and comprehensibility of CoCoSo, along with its skillful application of computation algorithms, are its main advantages^29^. Prior research typically evaluated the input components’ weights and percentage contributions exclusively. However, the weights of the output components play a vital and crucial role in the characteristic group executive. Thus, identifying and evaluating the significance of decision-making is an intriguing area of research. The aforementioned analysis of the literature indicates that the CoCoSo method’s applicability in uncertain decision-making environments can be further enhanced by combining it with grey theory. The advantage of the CoCoSo approach to making decisions is that it uses a combination and compromise percentage as an indicator, which ensures an internal balance of final utility during decision making and does not have compensatory issues that typical decision making has relatively low computational complexity^30–32^. An innovative model for sustainable supplier selection is introduced in this paper, which employs an enhanced CoCoSo fuzzy method in which the Bonferroni mean is incorporated to arrive at the weight derived from the fuzzy best and worst methods^33,34^. Scope of application of the CoCoSo Method: The development of an integrated MCDM technique for the evaluation of occupational health safety risk by applying the CoCoSo method in a fuzzy interference-based environment^35–37^.

Recently, multicriteria decision-making tools have also received significant attention in the 3D printing/additive manufacturing (AM) sectors. AM technology uses computer-aided design (CAD) models to 3D print complex geometries into models. A multicriteria decision-making tool was used to optimize the drilling process of 3D-printed polylactic acid polymers by considering seven machine learning algorithms. The top-performing machine learning algorithms in terms of predictive performance are random forest regressors, AdaBoost regressors, gradient boosting, and extremely randomized trees^38^. Özcan et al. studied MQL (minimum quantity lubrication) and N-MQL (multiwalled carbon nanotube-reinforced nanofluid) for milling CF/PEEK thermoplastic composites^39^. The cutting parameters determined by these scholars are the best for machining the CF/PEEK. The parameters applicable to the N-MQL cutting condition, i.e., a low level of cutting speed and low feed values, were inferred from multicriteria decision-making approaches. Kumar et al. explored the machining of polymer composite surfaces using jute fiber-reinforced materials^40^. In this study, different approaches are used to optimize process parameters, including the RSM, GRA, GA, and TLBO. The results show that the thrust force increases with increasing feed rates, decreasing spindle speed and decreasing drill diameter, whereas the surface roughness decreases with decreasing spindle speed, decreasing feed and decreasing drill diameter. Barik et al. implemented multicriteria decision-making methods, such as MOORA, TOPSIS, and VIKOR, for drilling process parameters and relationships among the parameters and defects of drilled holes^41^. The optimized drilling parameters led to a 20% reduction in top surface delamination defects but with 15% circularity error piercing and surface roughness, and a good correlation between TOPSIS and VIKOR was observed. This method effectively solves the drilling challenges of CFRP laminates. Thiru et al. studied hybrid composites based on woven E-glass fibers and expanded steel meshes into two stacking sequences via the MCDM method^42^. The experimental results indicated that the energy absorption and ductility of the spall are both increased when the strain increases to 204.5%. The best configuration was the vertically oriented composites containing fine steel fibers in the interior layer, with an overall percentage increase of 34.75%. Eisa et al. examined the cutting process parameters of CFRP products^43^. Optimization methods such as a regression model, a genetic algorithm and CoCoSo were employed to optimize every hole parameter. Significant improvements in hole quality were realized, including improvements of 29.57% at varying thicknesses.

From the aforementioned review of the literature, there is a need to develop an advanced and sustainable CoCoSo strategy. Thus, the current investigation aims at an approach that consists of a synergistic effect of GRA with D-CRITIC weights to arrive at optimal machining parameters for the application of drilling the CFRP plates. This study suggests the use of a novel grey extension of the CoCoSo approach called grey-CoCoSo. This research adds to and discusses the drilling of CFRPs and finds the optimal process parameters, particularly in the automobile and aerospace industries. The advantages of the two approaches are successfully synthesized in an implemented weight determination method based on the CRITIC and distance, which also improves the dependability of the produced weights of the criteria. A means of solving machining issues in an uncertain environment, known as the grey-coCoSo approach, is developed by combining the CoCoSo method with extended grey numbers. The suggested method is further used to determine the ideal process parameters for CFRP machining, thus suggesting the use and efficacy of the proposed approach.

## Materials and methodology

### CFRP

The workpiece used in the current investigation is a CFRP plate with dimensions of 150 × 150 × 5 mm. The plate is prepared by laminating several layers of prepregs with a thickness of 0.18 mm. The fibers are oriented at 0°, 45°, and 90°. The custom-designed drilling tool, made up of high-performance carbide, was used for drilling the CFRP plates.

### Design of experiments

**Fig. 1:**
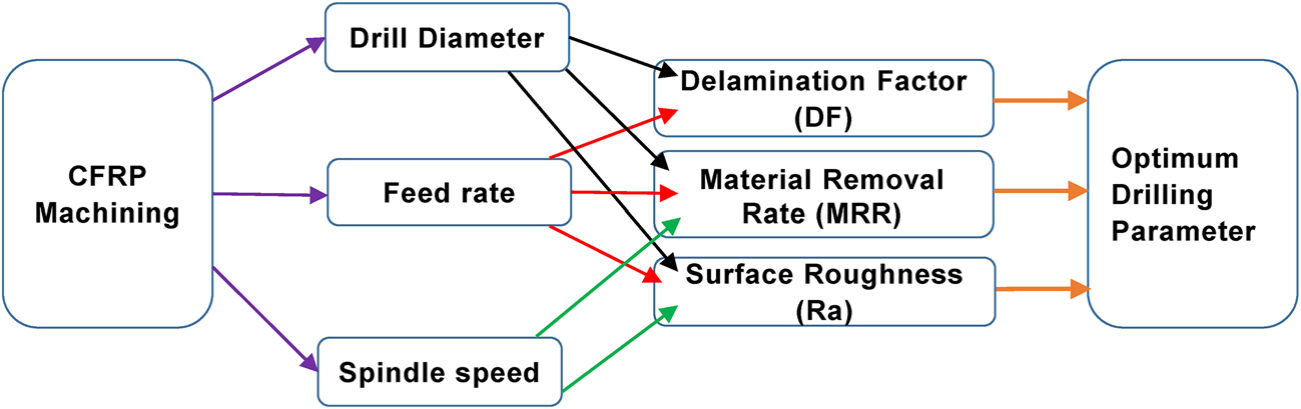
Framework of CFRP Machining.

The drilling experiments were carried out on a CNC machine (Make: Jyoti CNC Automation Limited, Model: nvu VMC 430). The spindle rotation was adjusted to 7500 rpm on the basis of the cutting tool supplier’s reference standard. There were three suggested feed rate levels—0.1 mm/rev, 0.2 mm/rev, and 0.3 mm/rev. Figure 1 presents the framework of the machining process used in the current study. The orthogonal array method was used to design the cutting parameters, with three levels for each parameter. Table 1 presents the specifics.

**Table 1:** Process parameters and their levels used for drilling.

Levels	Parameters		
	Spindle Speed (N) (rpm)	Feed Rate (F) (mm/rev)	Drill Diameter (D) (mm)
1	4500	0.1	4
2	6000	0.2	6
3	7500	0.3	8

DF and Ra were used to assess the quality of the holes. The Ra was measured using MAHR and MARSURF M400 equipment. DF was measured using a microscope (MF-A2017D, Mitutoyo, Sakado, Japan), and delamination was calculated using Eq. (1) on the basis of the ratio of the hole’s D_min_ to D_max_.

1 $$\:DF=\:\frac{{D}_{max}}{{D}_{min}}$$

The proposed methodology is based on the combination of two models—the exponentially weighted product model and the simple additive weighting model. It can be an assortment of workable middle ground. The CoCoSo decision problem is solved via the following steps, which are based on the estimation of the alternatives and relevant criteria^30–32^:

Step 1: Building a decision matrix using Eq. (2).

2 $$\:\text{X}\:=\:[{x}_{ij}{]}_{mXn\:\:}\:=\:\:\left[\begin{array}{ccc}{x}_{11}&\:\cdots\:&\:{x}_{1n}\\\:\vdots&\:\ddots\:&\:\vdots\\\:{x}_{m1}&\:\cdots\:&\:{x}_{mn}\end{array}\right]$$

where m is the number of options, n is the number of criteria, and x_ij_ is the value of criterion j at option i for i = 1, 2…, m, j = 1, 2…, n.

Step 2: Normalize the decision matrix.

In the present study, the cost‒profit normalization method was used. For the profit criteria, Eq. (3) was used, and for the cost criteria^32^, Eq. (4) was used.

3 $$\:{r}_{ij\:}=\:\frac{{x}_{ij}-min{\:x}_{ij}}{\text{max}{x}_{ij\:}-\text{min}{x}_{ij}}$$

4 $$\:{r}_{ij\:}=\:\frac{max{\:x}_{ij}-\:{x}_{ij}}{\text{max}{x}_{ij\:}-\text{min}{x}_{ij}}$$

Step 3: Calculation for the biased sum of the evaluation sequence and the total power weight of the comparison sequences for any substitute is S_i_ and P_i_ using Eqs. (5) and (6)^44^.

5 $$\:{S}_{i}=\:\sum\:_{j=1}^{n}{w}_{j}{r}_{ij}$$

6 $$\:{P}_{i}=\:\sum\:_{j=1}^{n}{{(\:r}_{ij})}^{{w}_{j}}$$

Step 4: Computation of the relative weights of alternatives using aggregation strategies via Eqs. (7)–(9).

7 $$\:{K}_{ia}=\:\frac{{P}_{i}+{S}_{i}}{{\sum\:}_{i=1}^{m}({P}_{i\:}+\:\:{S}_{i})}$$

8 $$\:{K}_{ib}=\:\frac{{S}_{i}}{{\text{min}S}_{i}}+\:\frac{{P}_{i}}{\text{min}{P}_{i}}$$

 

9 $$\:{K}_{ic}=\:\frac{\alpha\:\:{S}_{i}+(1-\alpha\:){S}_{i}}{(\alpha\:\text{max}{S}_{i}+\left(1-\alpha\:\:\right)\text{max}{P}_{i})} for{\text{ }}0{\text{ }} \leqslant {\text{ }}\alpha {\text{ }} \leqslant 1$$

Step 5: Determine the final ranking of the substitutes on the basis of the Ki values defined using Eq. (10). The higher the ki value is, the better the ranking.

10 $$\:{K}_{i}=({{K}_{ia}+{K}_{ib}+{K}_{ic})}^{\frac{1}{3}}+\:\frac{1}{3}\:({K}_{ia}+{K}_{ib}+{K}_{ic})$$

**Fig. 2:**
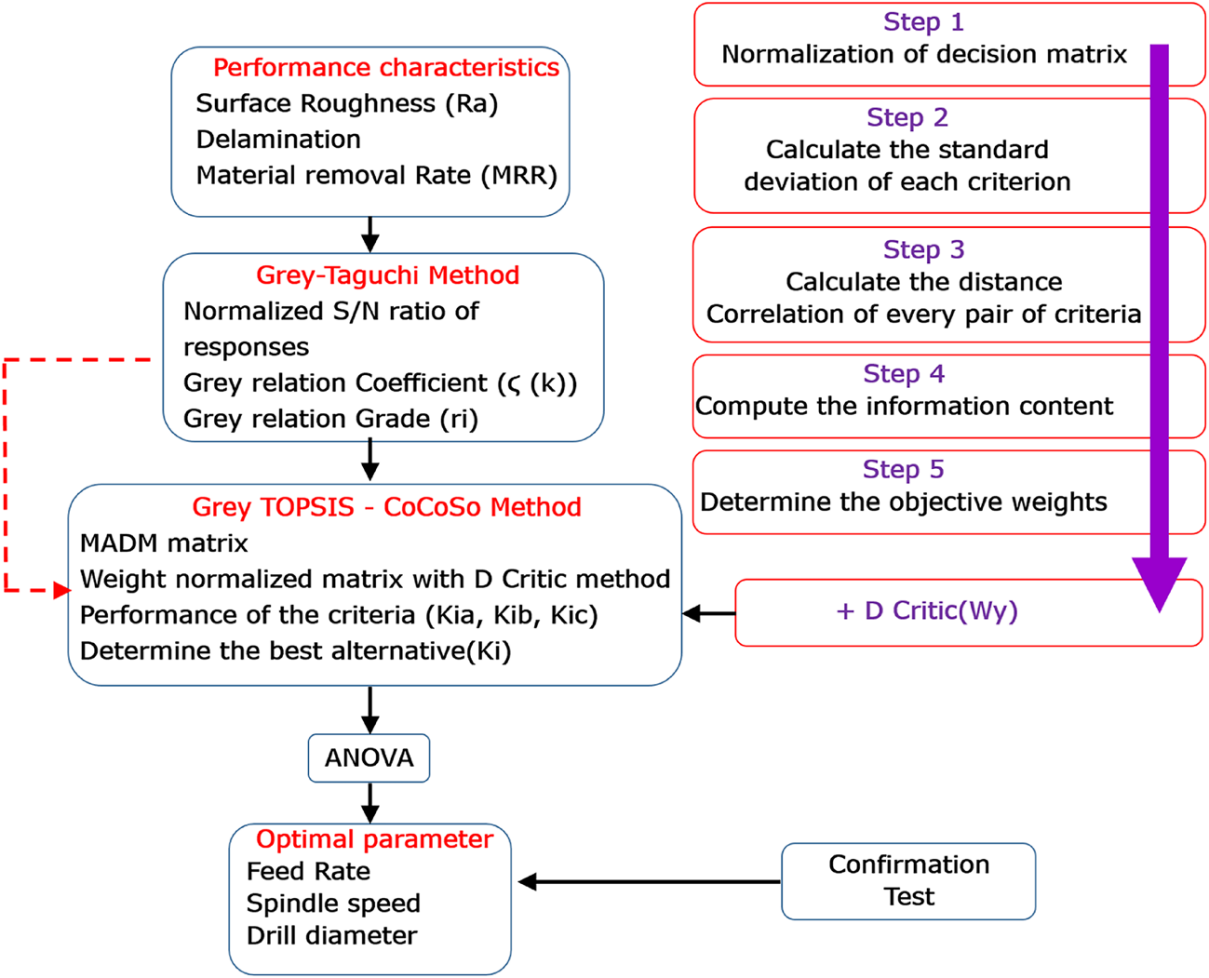
Flowchart of Process Parameter Optimization.

Combining several multicriteria optimization techniques saves time, simplifies data processing, and provides readers with a more effective way to select a suitable criterion. Furthermore, the multicriteria decision-making tool CoCoSo approach is employed to identify the ideal combination of drilling parameters. Later, the output drilling parameters estimated by the proposed combined use of CoCoSo and other criterion weight allocation methodologies are compared with the ideal drilling parameters. In addition, the estimation of the PCA-embedded CoCoSo method for multiobjective optimization of competing responses, such as thrust force (Th), torque (Tr), and surface roughness (Ra), is also highlighted in this study. Through the process of aggregation, the PCA tool effectively determines the answer priority weight. The purpose of the confirmatory test was to assess the effectiveness of the suggested hybrid module. The best parametric pairings for machining processes were found via an integrated application of the combined compromise solution (CoCoSo) and stepwise weight assessment ratio analysis (SWARA)^27,35^. The SWARA-CoCoSo method was found to perform better than other widely used optimization strategies in terms of determining the ideal parametric intermixes for machining operations that result in enhanced machining performance with the least amount of negative environmental impact. Consequently, in machining scenarios involving contradictory answers, this optimization technique might be used. The GRC was replaced with the hybrid approach in this study’s CoCoSo process to assess the optimum performance characteristics. Figure 2 shows the computational procedure.

### Weight estimation via the D-CRITIC technique

**Fig. 3:**
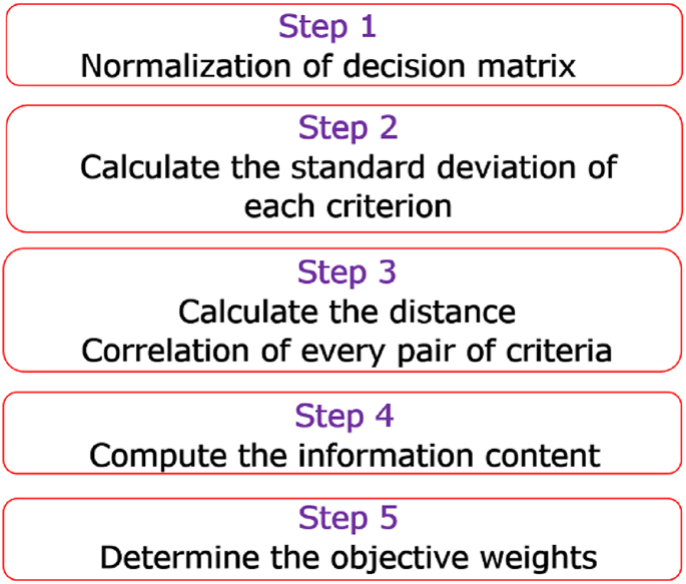
Steps followed to determine the weights in D-CRITIC.

By integrating the concept of distance correlation into the novel CRITIC approach, the suggested D-CRITIC technique was created^32^. Overall, five essential phases are involved in applying D-CRITIC, as illustrated in Fig. 3. The subsequent sections offer a thorough breakdown of every stage.

Step 1 is the normalization of the choice matrix. Since the scores of several criteria are expressed in disparate scales or measurement units, they cannot be compared. The normalization process involves converting the scores into standard scales, with a range of 0–1. The first step in the suggested approach is to normalize the available scores in the choice matrix using Eq. (11).

11 $$\:\stackrel{-}{{x}_{ij}}=\:\frac{{x}_{ij}-{x}_{jmin}}{{x}_{jmax}-{x}_{jmin}}$$

where $\:\stackrel{-}{{x}_{ij}}$ is the alternative’s normalized score with respect to criterion j, $\:{x}_{ij}$ is the alternative’s real score with respect to criterion j, $\:{x}_{jmax}$ denotes the criterion’s best score, and $\:{x}_{jmin}$ denotes the criterion’s worst score.

Step 2 is to determine each criterion’s standard deviation (SD_j_) using Eq. (12).

12 $$\:{SD}_{j}=\sqrt{\frac{{\left(\sum\:_{i=1}^{n}{x}_{ij}-\stackrel{-}{{x}_{j}}\right)}^{2}}{n-1}}$$

Where m is the total number of possibilities, $\:{x}_{ij}$ represents the mean score of measure j, n is the total number of potential answers, and $\:\stackrel{-}{{x}_{j}}$ is the average score of measure j.

Step 3 determines each pair of criteria’s distance correlation, which shows the primary distinction between the original CRITIC approach and the suggested D-CRITIC method. The Pearson correlation is used in the original CRITIC technique to identify the contradictory correlations between criteria. However, as mentioned earlier, there is a chance that the Pearson correlation will not adequately represent the true relationships between the measures. More precisely, there is a possibility that two criteria with a zero Pearson relationship coefficient are not entirely independent. Consequently, it follows that the distance correlation—a novel correlation measure introduced by Székely et al.^30^—is zero only in the case of independent criteria. To minimize the amount of error in the resulting weights, the distance relationship is used as an additional method for modelling the dependencies in the modified D-CRITIC approach. The distance relationship between cj and cj0 is defined by Eq. (13).

13 $$\:dCorr\left({C}_{1j,}{C}_{2j}\right)=\frac{dCorr\left({C}_{1j,}{C}_{2j}\right)}{\sqrt{dVar\left({C}_{1j}\right)dVar\left({C}_{2j}\right)}}$$

where $\:dCorr\left({C}_{1j,}{C}_{2j}\right)$, is the distance covariance of $\:{C}_{1j\:}$ and ${C}_{2j}$, and $\:dVar\left({C}_{1j}\right)$ = $\:dCorr\left({C}_{1j,}{C}_{1j}\right)$ is the distance variance of $\:{C}_{1j}$. Additionally, $\:dVar\left({C}_{2j}\right)$ = $\:dCorr\left({C}_{2j,}{C}_{2j}\right)$ is the distance covariance of $\:{C}_{2j}$. The process of ascertaining the distance relationship between each of the two criteria, $\:{C}_{1j\:}$ and ${C}_{2j}$, is executed as per the steps (3.1–3.5) mentioned below:

Step 3.1: Using the scores connected to each of the options being evaluated, create the Euclidean distance matrix of $\:{C}_{1j}$ and create a matrix that is comparable to $\:{C}_{2j}$.

Step 3.2: Double-center each matrix by carrying out the subsequent steps until the elements’ overall means, row means, and column means are all zero: subtract the row mean from each element; add the matrix mean to each element in the output; then, subtract the column mean from each element.

Step 3.3: Multiply the elementwise double-centered matrices and determine the average element value from the resultant matrix, or the sum of the components split by the total number of components. $\:dCorr\left({C}_{1j,}{C}_{2j}\right)$, or the distance covariance of $\:{C}_{1j\:}$ and ${C}_{2j}$, is the square root of this mean value.

Step 3.4: Calculate the distance alteration of $\:{C}_{1j}$, $\:dVar\left({C}_{1j}\right)$and the distance variance of $\:{C}_{2j}$ 0, $\:dVar\left({C}_{2j}\right)$. As $\:dVar\left({C}_{1j}\right)$ = $\:dCorr\left({C}_{1j,}{C}_{1j}\right)$ and $\:dVar\left({C}_{2j}\right)$ = $\:dCorr\left({C}_{2j,}{C}_{2j}\right)$, these two values may be calculated by re-running Steps 3.1–3.4.

Step 3.5: The distance correlation between $\:{C}_{1j\:}$ and $\:{C}_{2j}$or $\:dCorr\left({C}_{1j,}{C}_{2j}\right)$, is found by substituting the available $\:dCorr\left({C}_{1j,}{C}_{2j}\right)$ in Eq. (14).

Step 4 determines the content of the information in criterion j using Eq. (14), where the information content is indicated by $\:{I}_{j}$.

14 $$\:{I}_{j}={SD}_{j}\sum\:_{2j=1}^{n}\left(1-dCorr\left({C}_{1j,}{C}_{2j}\right)\right)$$

Step 5 establishes the target weights using Eq. (15) by estimating the objective weight of criterion j, where $\:{W}_{j}$ is the objective weight

15 $$\:{W}_{j}=\frac{{I}_{j}}{\sum\:_{1j=1}^{n}{I}_{j}}$$

Later, the GRA is executed. The first stage in the GRA process is data preprocessing, which normalizes the random grey data using various measurement units to create dimensionless parameters using Eqs. (16) and (17). The original data are preprocessed on the basis of the quality characteristics of the sequence data.

16 $$\:{x}_{i}=\frac{{y}_{i}\left(k\right)-\text{min}\left({\:y}_{i}\left(k\right)\:\right)}{\text{max}\left({\:y}_{i}\left(k\right)\:\right)-\:\text{min}\left({\:y}_{i}\left(k\right)\:\right)}$$

17 $$\:{x}_{i}=\frac{\text{max}\left({\:y}_{i}\left(k\right)\:\right)-{y}_{i}\left(k\right)}{\text{max}\left({\:y}_{i}\left(k\right)\:\right)-\text{min}\left({\:y}_{i}\left(k\right)\:\right)}$$

For each of the collected deviational sequencing data separately, the grey relational coefficient ξ_i_(k) is determined using Eq. (18).

18 $$\:{\xi\:}_{i}\left(k\right)=\frac{{{\Delta\:}}_{\text{m}\text{i}\text{n}}+\varPsi\:{{\Delta\:}}_{\text{m}\text{a}\text{x}}}{{{\Delta\:}}_{0\text{i}}\left(k\right)+\varPsi\:{{\Delta\:}}_{\text{m}\text{a}\text{x}}}$$

Later, the relational grade after the grey relational coefficient in the final phase is obtained, is calculated using Eq. (19), where n indicates the number of process responses.

19 $$\:\gamma\:\left(i\right)=\frac{1}{n}{\sum\:}_{k=1}^{n}{\xi\:}_{i}\left(k\right)$$

## Results and discussions

### GRA

**Fig. 4:**
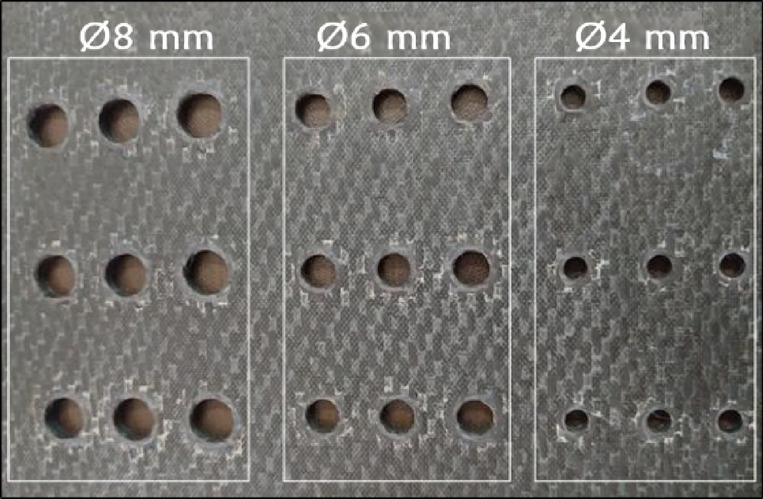
CFRP plate drilled with 8, 6, and 4 mm diameter holes.

The results obtained after the preceding phase condition the GRA. First, normalization is carried out via the smaller-is-better (Ra & DF) and larger-is-better (MRR) criteria, which are based on the aim of the response output (Eqs. 16 and 17). Next, the computation of deviation from the relation list was carried out. The grey correlation coefficient (GRC) for every experiment was then calculated (Eq. 18). Equations were then used to obtain the average value of the GRG for the Ra, DF, and MRR (Eq. 19). The drilling experiments were carried out on the milling machine (Make: Jyoti CNC Automation Limited, Model: nvu VMC 430). Figure 4 shows the CFRP plate drilled with holes of various diameters— 8, 6, and 4 mm. Table 2 presents an example of the outcome.

**Table 2:** Experimental results and the GRA.

Sl. No	Input Parameters			Output Responses			Grey Relational Coefficient			Weighted GRG	Rank
	N	F	D	Surface Roughness (Ra) (µm)	Delamination Factor (DF)	MRR (gm/min)	Ra	DF	MRR		
1	4500	0.1	4	3.650	1.043	0.053	0.572	0.520	0.358	0.483	17
2	4500	0.1	6	3.600	1.042	0.055	0.595	0.536	0.362	0.498	14
3	4500	0.1	8	3.830	1.045	0.056	0.502	0.495	0.367	0.454	18
4	4500	0.2	4	3.770	1.049	0.050	0.523	0.448	0.348	0.440	19
5	4500	0.2	6	3.800	1.056	0.051	0.512	0.391	0.351	0.418	22
6	4500	0.2	8	3.900	1.049	0.053	0.479	0.451	0.355	0.428	20
7	4500	0.3	4	4.440	1.064	0.047	0.354	0.340	0.339	0.345	26
8	4500	0.3	6	4.330	1.063	0.046	0.374	0.343	0.336	0.351	25
9	4500	0.3	8	4.570	1.065	0.045	0.333	0.333	0.333	0.333	27
10	6000	0.1	4	3.430	1.038	0.064	0.690	0.590	0.397	0.559	9
11	6000	0.1	6	3.250	1.040	0.067	0.831	0.563	0.408	0.600	7
12	6000	0.1	8	3.400	1.039	0.076	0.710	0.582	0.450	0.581	8
13	6000	0.2	4	3.600	1.045	0.058	0.595	0.493	0.375	0.487	15
14	6000	0.2	6	3.470	1.044	0.060	0.665	0.507	0.383	0.518	12
15	6000	0.2	8	3.800	1.041	0.063	0.512	0.547	0.392	0.484	16
16	6000	0.3	4	4.020	1.049	0.053	0.444	0.446	0.358	0.416	23
17	6000	0.3	6	3.990	1.050	0.055	0.452	0.439	0.364	0.419	21
18	6000	0.3	8	4.040	1.052	0.057	0.439	0.424	0.371	0.411	24
19	7500	0.1	4	3.200	1.028	0.099	0.880	0.846	0.622	0.783	3
**20**	**7500**	**0.1**	**6**	**3.100**	**1.024**	**0.118**	**1.000**	**1.000**	**1.000**	**1.000**	**1**
21	7500	0.1	8	3.300	1.027	0.123	0.786	0.892	0.890	0.893	2
22	7500	0.2	4	3.300	1.027	0.085	0.786	0.862	0.506	0.718	5
23	7500	0.2	6	3.240	1.027	0.093	0.840	0.874	0.560	0.758	4
24	7500	0.2	8	3.480	1.032	0.101	0.659	0.716	0.642	0.672	6
25	7500	0.3	4	3.770	1.034	0.071	0.523	0.669	0.427	0.540	10
26	7500	0.3	6	3.900	1.034	0.076	0.479	0.685	0.453	0.539	11
27	7500	0.3	8	4.050	1.039	0.082	0.436	0.578	0.486	0.500	13

**Fig. 5:**
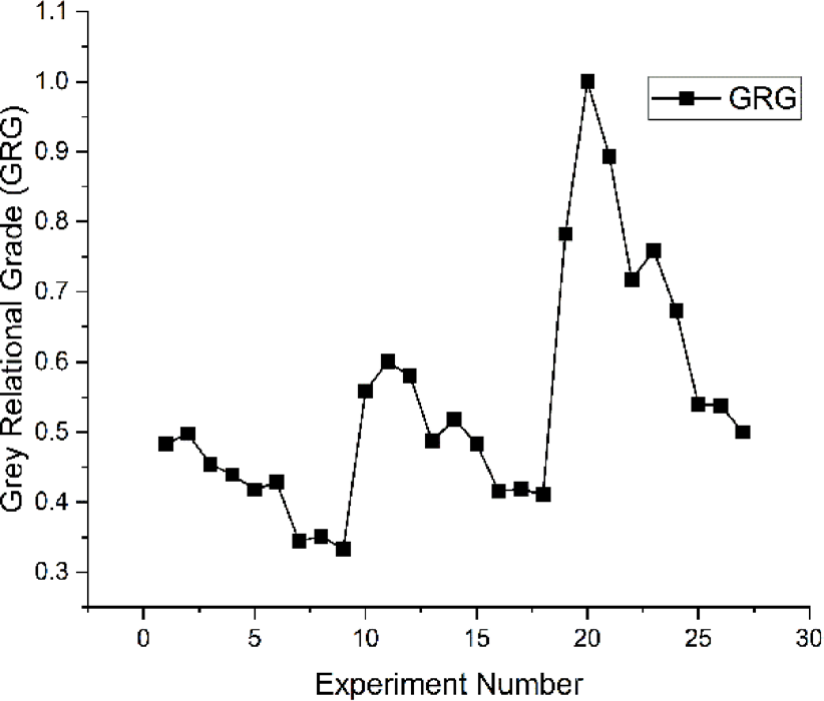
Experimental Trials vs. GRG.

Table 3 shows that the drilling parameter of the 20^th^ trial with a grey relation grade of 1 is the greatest, and the GRG with the highest value is always desirable. This finding is in good agreement with the depiction in Fig. 5. Consequently, out of the 27 experiments, the 20^th^ experiment produced the best multiresponse output.

**Fig. 6:**
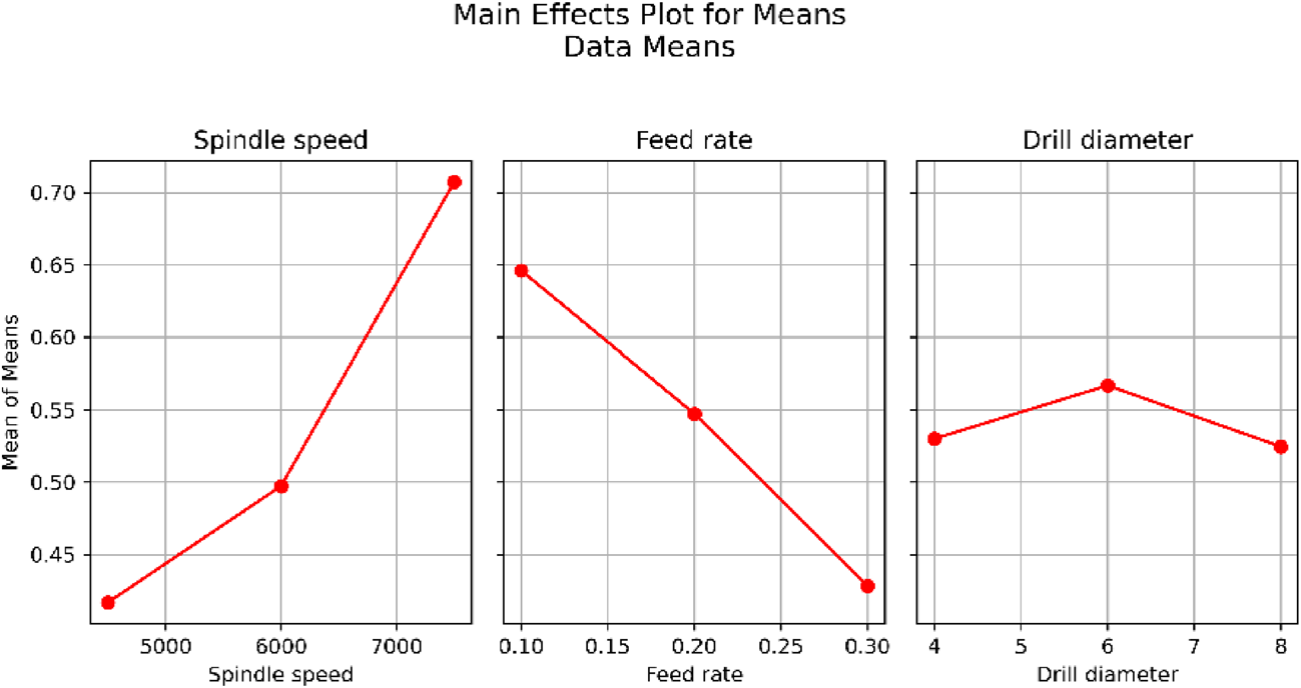
GRG Plot.

The GRG is used to rank the correlation between the consultation and referenced series. The referenced series and the consultation series have a significant correlation, as indicated by the greater GRG. For each attribute N, F, and D, the ideal drilling parameter restrictions are determined via signal-to-noise (S/N) ratio analysis. The ideal combination of drilling parameters was found at N3, F1, and D2, which is the result for the S/N ratios of the GRG in Fig. 6. The response table for the means, which details the delta values of the drilling process parameters, is displayed in Table 3. At a feed rate of 0.1 mm/rev, a high spindle speed of 7500 rpm, and a drill diameter of 6 mm, the drill quality was noted.

**Table 3:** GRG response.

Levels	Parameters		
	Spindle Speed (N) (rpm)	Feed Rate (F) (mm/rev)	Drill Diameter (D) (mm)
1	−7.687	−4.097	−5.776
2	−6.152	−5.451	−5.392
3	−3.213	−7.504	−5.884
Delta	4.475	3.407	0.492
Rank	1	2	3

### D-CRITIC

In this context, an attempt has been made to reflect the suitability of the D-CRITIC method, an objective weighing method, as an alternative method to identify the ranks and weights of the performance criteria of the drilling process. There are 27 options and 3 criteria in the decision problem. The remaining two components, Ra (C1) and DF (C2), are designated cost-based factors. The requirements (C3), the MRR, are designated a profit-based factor. Initially, the distance correlation measurements of the criteria were calculated, and the distance correlation matrix of the criteria is shown in Table 4. The data and specifics for the CFRP drilling, including the weights of the decision criteria, are displayed in Table 5. Table 5 contains the data on CFRP drilling as well as the weights assigned to each criterion to assess the machining performance characteristics.

**Table 4:** Distance Cor-Matrix.

Criterion	Ra	DF	MRR
Ra	1	0.85291	0.69295
DF	0.85291	1	0.85844
MRR	0.69295	0.85844	1

**Table 5:** Weights assigned to each criterion.

Criteria	C1	C2	C3
Weights	0.36559	0.24906	0.38535
Optimal Value	Min.	Min.	Max.

### CoCoSo

In the earlier section, Eqs. 3 and 4 are used to form the normalized decision-making matrix. This is followed by the formation of the comparability sequence matrix. The algorithm in this procedure incorporates the weights of the decision-making criterion. With Table 6, one must use Eqs. (5) and (6) to produce the Si and Pi vectors, respectively. Table 6 presents the values that were obtained. Aggregation procedures are used to produce the final ranking results. At this point, Eqs. (7), (8), and (9) are used to obtain the values of Kia, Kib, and Kic, respectively. These Ki values serve as the basis for ranking the choices. The ranking score by K is obtained using Eq. (10) to obtain the alternatives’ final ranks.

**Table 6:** Comparability sequence matrix along with S_i_ and P_i_.

Exp. No	C1	C2	C3	S_i_	C1	C2	C3	P_i_
1	0.229	0.134	0.039	0.402	0.843	0.857	0.415	2.114
2	0.241	0.141	0.046	0.429	0.859	0.869	0.441	2.169
3	0.184	0.122	0.053	0.359	0.778	0.837	0.466	2.081
4	0.199	0.096	0.024	0.318	0.801	0.788	0.342	1.931
5	0.191	0.055	0.030	0.276	0.789	0.687	0.373	1.849
6	0.167	0.097	0.036	0.300	0.750	0.792	0.401	1.943
7	0.032	0.008	0.010	0.050	0.412	0.419	0.245	1.077
8	0.060	0.011	0.000	0.071	0.516	0.459	0.000	0.975
9	0.000	0.000	0.005	0.005	0.000	0.000	0.188	0.188
10	0.284	0.162	0.093	0.539	0.911	0.899	0.577	2.387
11	0.328	0.152	0.106	0.586	0.961	0.885	0.608	2.454
12	0.291	0.159	0.150	0.601	0.920	0.895	0.696	2.511
13	0.241	0.121	0.064	0.426	0.859	0.835	0.500	2.194
14	0.274	0.128	0.075	0.476	0.899	0.847	0.531	2.278
15	0.191	0.146	0.086	0.424	0.789	0.876	0.562	2.227
16	0.137	0.094	0.040	0.271	0.698	0.785	0.417	1.900
17	0.144	0.090	0.049	0.283	0.712	0.776	0.451	1.939
18	0.132	0.080	0.059	0.270	0.689	0.753	0.484	1.926
19	0.341	0.226	0.268	0.835	0.975	0.976	0.870	2.821
20	0.366	0.249	0.362	0.976	1.000	1.000	0.976	2.976
21	0.316	0.234	0.385	0.935	0.948	0.985	1.000	2.932
22	0.316	0.229	0.198	0.742	0.948	0.979	0.773	2.700
23	0.331	0.231	0.234	0.796	0.964	0.982	0.825	2.771
24	0.271	0.200	0.278	0.749	0.896	0.946	0.882	2.724
25	0.199	0.187	0.127	0.514	0.801	0.932	0.652	2.385
26	0.167	0.192	0.152	0.511	0.750	0.937	0.699	2.387
27	0.129	0.158	0.181	0.469	0.684	0.893	0.748	2.325

Table 7 shows how this vector is represented. On the basis of Table 7, the optimal machining parameter is found in experiment number 20, and the poorest option is found in experiment number 9. When one ensures that the final Ki and the rankings generated by each component of Ki are in maximum agreement, this method is completely reliable^31^. The observation indicates that every individual ranking and the final ranking are identical. Thus, the following conclusions can be drawn for CFRP drilling: 20 > 21 > 19 > 24 > 23 > 22 > 12 > 11 > 10 > 25 > 26 > 14 > 27 > 2 > 13 > 15 > 1 > 3 > 4 > 6 > 17 > 16 > 5 > 18 > 8 > 7 > 9.

As indicated in Table 7, for all the experimental trials, the order of the K_i_ scores is decreased to determine the final ranking. Trial numbers 20 and 21 appear to yield the best performance in terms of aggregate values and CoCoSo rankings for all three process parameters. This finding indicates that the optimal drilling setup for enhanced performance is in accordance with the measured performance characteristics. On the other hand, the worst performer is trial nos. 9 and 7 for all three process parameters, producing very low values of K_ia_ = 0.003, K_ib_ = 2.0, and K_ic_ = 0.049, leading to the worst possible overall rank. The highest rank of 1 was achieved by experiment number 20, with higher values in K_ia_, K_ib_, and K_ic_. The optimized process parameter values of N, F, and D are 7500 rpm, 0.1 mm/rev, and 6 mm, respectively. This ranking approach means that each of the experimental factors is assessed, and the best setup that leads to the highest performance is determined. The symbol K_i_ represents the most favourable rating result for each factor across all the experiments performed.

**Table 7:** Aggregate values and CoCoSo ranking.

Exp. No	K_ia_	Rank	K_ib_	Rank	K_ic_	Rank	K_i_	Rank
1	0.036	17	185.871	17	0.637	17	67.912	17
2	0.037	16	191.958	14	0.657	16	70.007	14
3	0.034	18	174.610	18	0.617	18	64.037	18
4	0.032	19	164.117	19	0.569	19	60.412	19
5	0.030	24	146.983	23	0.538	24	54.494	23
6	0.032	20	158.416	20	0.568	20	58.448	20
7	0.016	25	41.323	26	0.285	25	17.365	26
8	0.015	26	58.233	25	0.265	26	23.420	25
9	0.003	27	2.000	27	0.049	27	1.949	27
10	0.041	9	215.841	9	0.740	9	78.219	9
11	0.043	8	224.675	8	0.769	8	81.250	8
12	0.044	7	231.616	7	0.787	7	83.634	7
13	0.037	15	190.926	15	0.663	15	69.656	15
14	0.039	13	201.932	12	0.697	13	73.441	12
15	0.037	14	190.369	16	0.671	14	69.468	16
16	0.031	23	148.460	22	0.549	23	55.008	22
17	0.031	21	151.681	21	0.562	21	56.124	21
18	0.031	22	145.697	24	0.556	22	54.056	24
19	0.048	3	289.822	3	0.925	3	103.550	3
20	0.054	1	355.634	1	1.000	1	125.986	1
21	0.051	2	346.263	2	0.979	2	116.042	2
22	0.045	6	261.141	6	0.871	6	93.745	6
23	0.047	4	276.092	5	0.903	4	98.859	5
24	0.048	5	277.956	4	0.879	5	99.497	4
25	0.041	10	210.560	10	0.733	10	76.410	10
26	0.041	11	208.948	11	0.733	11	75.859	11
27	0.039	12	198.838	13	0.707	12	72.387	13

**Fig. 7:**
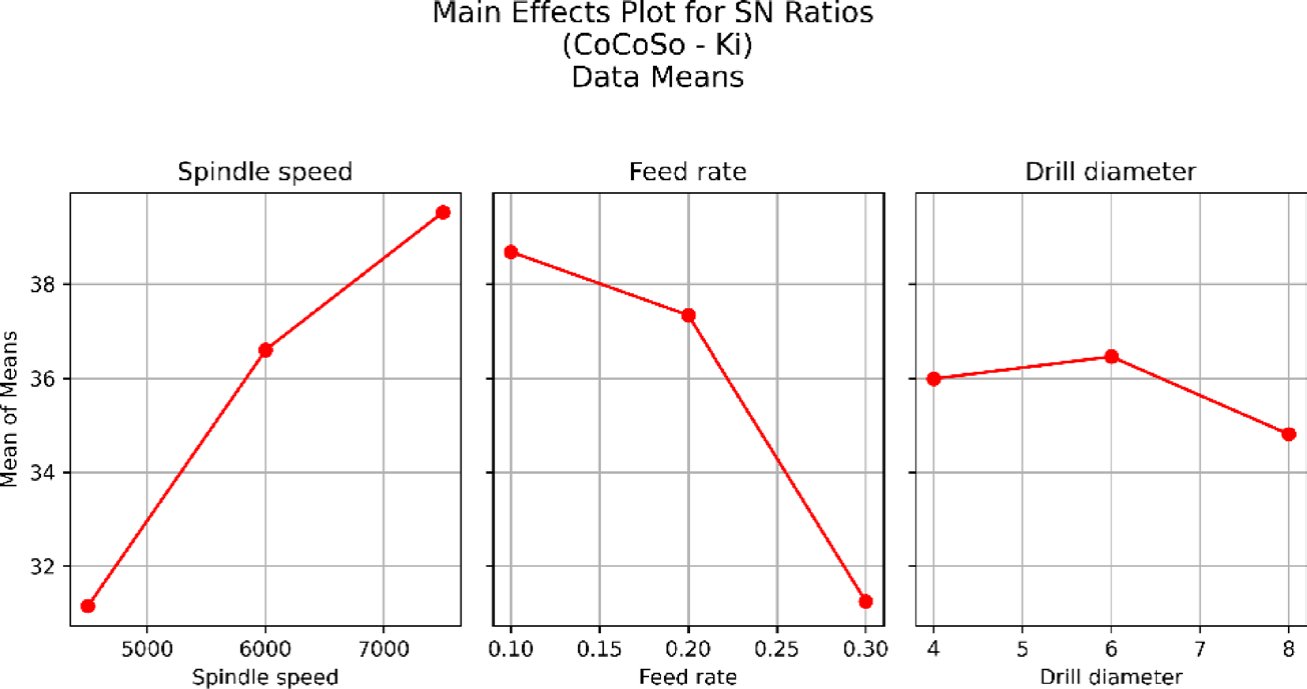
S/N Ratio of CoCoSo (K_i_).

Table 8 shows that the ideal parametric combination for Ra = 3.1 μm, DF = 1.024, and MRR = 0.118 gm/min is provided by experimental run number 20, which has *N* = 7500 rpm, F = 0.1 mm/rev, and D = 6 mm. Furthermore, for the specified drilling operation, trial 9 is the least favourable combination of input parameters, whereas trial 21 is the next best parametric combination. Importantly, the ideal experimental run and its composite score are presented in trial 20 in Table 8. The major effects plot shown in Fig. 7 shows that identical optimal levels are achieved by both the S/N ratio information and the raw information. From Fig. 7, it is evident that higher spindle speeds result in better performance in terms of the S/N ratio, which typically indicates a more stable and efficient process with less variation. This implies that the spindle speed is a significant factor in refining the excellence or performance of the drilling process. The relationship between the mean S/N ratio and F appears to be flat, indicating that the feed rate has a minimal effect on the S/N ratio within the tested range. The drill diameter and S/N ratio appear to have a moderate relationship; however, they appear to follow a decreasing trend. A better performance was achieved by a 6 mm diameter drill, which results in a better balance between the signal and noise.

**Table 8:** Response of CoCoSo (K_i_).

Levels	Parameters		
	Spindle Speed (N) (rpm)	Feed Rate (F) (mm/rev)	Drill Diameter (D) (mm)
1	30.37	**38.66**	36
2	36.66	37.33	**36.52**
3	**39.5**	30.54	34.01
Delta	9.13	8.12	2.5
Rank	1	2	3

**Table 9:** Analysis of Variance – CoCoSo (K_i_).

Source	DF	Adj N	Contribution	Adj MS	F-Value	P-Value
N	2	11178.3	55.52	5589.14	1525.5	0
F	2	7719.5	38.34	3859.75	1053.48	0
D	2	82	0.41	41.01	11.19	0.005
N × F	4	807	4.01	201.76	55.07	0
N × D	4	178.3	0.89	44.57	12.17	0.002
F × D	4	138.3	0.69	34.58	9.44	0.004
Error	8	29.3	0.15	3.66		
Total	26	20132.7	100.00			
R^2^	99.85%					
R^2^ (Predicted)	98.34%					

The influencing critical degree of the process parameter needs to be confirmed. Hence, analysis of variance (ANOVA) was used to confirm the influence on the multiresponse yield during the machining of CFRPs. The specified significance level and confidence interval were 0.05 and 0.95, respectively. Table 9 shows that F, N, and D are noteworthy variables. The combination of N×F, N×D, and F×D also had a noteworthy value. With a contribution ratio of 55.52%, N had the highest value. According to the desired criterion output, the spindle speed therefore had the greatest influence on the drilling hole’s quality. The feed rate and drill diameter contributed 38.34% and 0.41%, respectively. The measured R^2^ value of 99.85% and the predicted R^2^ value of 98.34% indicate that the linear regression model is the best fit.

### Validation

The enhanced quality of the output response is confirmed by a verification trial. Equation (20) was used to calculate the expected optimal value.

20 $$\:{\eta\:}_{pred}={\eta\:}_{mean}+\sum\:_{i=1}^{n}\left({\eta\:}_{i}-{\eta\:}_{mean}\right)$$

where n is the number of parameter inputs and is equal to three in this case study, $\:{\eta\:}_{mean}\:$is the total mean value of the answer, and $\:{\eta\:}_{i}\:$is the average of the response at the optimal level.

The tool provider provided Table 10 with the following initial parameters: N = 4500 rpm, F = 0.1 mm/rev, and D = 4 mm. The same optimum values were obtained using the ideal parameters N, F, and D of 7500 rpm, 0.1 mm/rev, and 6 mm, respectively. These ideal values were estimated using GRA and Grey-CoCoSo. The results indicate that an enhanced assessment coefficient is found using the grey-co-co-so approach compared with the GRA method. Employing this suggested strategy clearly increased the quality and responsiveness of the CFRP drilling operation.

**Table 10:** Validation Results.

Parameters	Initial	Optimum Combination			
		GRA		Grey-CoCoSo	
		Predicted	Actual	Predicted	Actual
Level	N1, F1, D1	N3, F1, D2	N3, F1, D2	N3, F1, D2	N3, F1, D2
Ra	3.65		3.1		3.1
DF	1.043		1.024		1.024
MRR	0.053		0.118		0.118
K_i_	105.82			123.504	125.986
GRG	0.483	0.954	1		
Improvement		0.471	0.517	17.684	20.166

## Conclusions

A thorough literature review revealed that the CoCoSo method’s applicability in uncertain decision-making environments can be further enhanced by combining it with grey theory. In this context, the current investigation aims to use an enhanced CoCoSo strategy based on the grey coefficient with the contribution of the D-CRITIC weights and proposes a novel approach to identify the best parameter of the CFRP drilling process. Such attempts have rarely been reported in the literature. This study suggests the use of a novel grey extension of the CoCoSo approach called Grey-CoCoSo. This research adds to and discusses the drilling of CFRPs and finds the optimal process parameters, particularly in the automobile and aerospace industries. The advantages of the two approaches are successfully synthesized in an implemented weight determination method based on the CRITIC and distance, which also improves the dependability of the produced weights of the criteria.

Numerous studies have examined the relationship between machining parameters and drill diameter in relation to a specific component of CFRP composite machining technology. To solve parametric optimization challenges for the dry drilling of CFRP composite processes and create a sustainable manufacturing environment, this study suggests the integrated use of the Grey and CoCoSo methodologies. A thorough examination allows for the deduction of the following conclusions.

- The D-CRITIC technique assigns distinct weight sets to the examined responses, taking into account the differing needs of various stakeholders, as opposed to giving equal weights to all of them. This could result in various parametric intermixes to fully explore the process’s machining capabilities.
- The largest parameters that minimize Ra, DF, and maximum MRR were specified using the closest coefficient value. N3, F1, and D2 (high spindle speeds of 7500 rpm, 0.1 mm/rev feed rate, and 6 mm drill diameter, respectively) yielded the best results, with the lowest Ra values (3.1 μm) and 1.024 DF and the largest MRR (0.118 gm/min).
- The influence of the distribution of the drilling process input parameters was verified via ANOVA. The spindle speed, feed rate, and drill diameter contributed 55.52%, 38.34%, and 0.41%, respectively, of the total.
- For the D-CRITIC weight set, the ideal parametric conditions would be N = 7500 rpm, F = 0.1 mm/rev, and D = 6 mm. This would lead to improvements in the K_i_ value of 17.684% over the initial observation.
- Under realistic circumstances, the results of the analysis could be applied to the drilling operation to attain the required response quality and MRR. The findings show that the suggested approach can help with the MCDM problem in ambiguous and uncertain situations. Compared with other MCDM approaches, this reasonably sophisticated approach is a wonderful, helpful tool and is particularly effective in rejecting process variance.
- Every response is given a weight, which reduces the flexibility of the decision-making (DM) process. The method’s productivity may be further enhanced and applied to a sizable number of multicriteria inputs and response outputs. It is possible to expand the experimental approach to include other drilling parameter operations and assessments. Moreover, additional machining processes could benefit greatly from the application of this optimization technique.
- MCDM is a growing field of decision-making theory for experts of all kinds. Additionally, the advantage of the CoCoSo-GRA approach in making decisions is that it uses a combination and compromise percentage as an indicator, which ensures an internal balance of final utility during decision-making and does not at all have compensatory issues that typical decision-making has relatively low computational complexity.

Further investigations may be carried out to determine the relative significance of the output response using alternative subjective criterion weighting methods. Techniques such as the best‒worst approach and pivot pairwise relative criteria importance assessment (PIPRECIA) can be a great choice. In the future, the grey method may be used to solve parametric optimization problems for environmentally friendly and sustainable machining processes in conjunction with other, less well-known MCDM techniques, such as multiattributive real-ideal comparative analysis (MARICA) and multiattributive border approximation area comparison (MABAC). In addition, the consideration of other process parameters, such as tool material and tool geometry, can also be important for more realistic predictions and optimizations. Additionally, microscopic examination of machined samples using the optimized parameters of the presented newer technique has great potential.

## Data Availability

Data is provided within the manuscript or supplementary information files.
